# Novel roseophages provide insights into a genetically and ecologically diverse phage family

**DOI:** 10.1099/mgen.0.001568

**Published:** 2025-11-19

**Authors:** Zuqing Wu, Ying Wu, Chunmei Deng, Hang Xiao, Sige Wang, Songlin Ye, Yanlin Zhao, Zefeng Zhang

**Affiliations:** 1 Fujian Provincial Key Laboratory of Agroecological Processing and Safety Monitoring, College of JunCao Sciences and Ecology (College of Carbon Neutrality), Fujian Agriculture and Forestry University, Fuzhou, PR China

**Keywords:** CHUG, ecological distribution, genome sequence, *Roseobacter* phage

## Abstract

Roseobacters are prevalent in marine environments and play a crucial role in global carbon and sulphur cycles. Although many roseophages that infect roseobacters have been characterized, those infecting members of the ecologically dominant pelagic *Roseobacter* cluster (PRC) remain largely unexplored due to the challenges of culturing these organisms. In this study, we isolated 7 phylogenetically related roseophages from 3 PRC lineages and retrieved 279 uncultured viral genomes (UViGs) related to these roseophages from marine environmental viral databases. Comparative genomic and phylogenetic analyses revealed that these roseophages and their related UViGs form a novel family-level phage group (designated the CRP-822-type group) comprising at least five subgroups. These subgroups display distinct genomic features in terms of G+C content, amino acid usage and codon usage, suggesting host-range specialization. Host prediction suggests that subgroup V with low G+C content may infect the SAR86 clade, while the high G+C subgroup IV likely infects the high G+C KI89A clade. Finally, viromic read-mapping analyses revealed that CRP-822-type phages are widely distributed across the global ocean and are adapted to diverse marine environments. All members of subgroup IV were more abundant in trade, westerlies and coastal regions with high temperatures. The other four subgroups exhibited more divergent biogeographic patterns, with some members more abundant in trade and westerlies ocean regions, whereas others dominated in polar or estuarine regions. Collectively, this study elucidates the genetic diversity and ecology of a previously unrecognized marine phage group that infects PRC roseobacters and other important marine bacteria.

**Impact Statement**

Understanding the diversity and ecological roles of roseophages is critical for elucidating their impact on the metabolism and evolution of roseobacters. However, studies on roseophages, especially those infecting pelagic *Roseobacter* cluster (PRC) members, remain limited. By isolating and characterizing 7 novel phages infecting 3 PRC lineages and retrieving 279 related UViGs, this study identifies a novel family-level phage group designated CRP-822-type. The CRP-822-type phages are ubiquitous across diverse marine environments and are adapted to varying environmental conditions, such as temperature and salinity. Collectively, this work significantly expands our understanding of roseophage diversity, establishes a new host-phage interaction mode and characterizes a novel phage group.

## Data Summary

The genome sequences of the seven roseophages, all CRP-822-type UViGs and the scripts and source data for the figures are publicly available on Figshare (https://doi.org/10.6084/m9.figshare.30171130). The seven roseophage genomes have also been deposited in the GenBank database under accession numbers PV211008-PV211014. The 16S rRNA gene sequences of FZCC1911 and FZCC0197 have been deposited in GenBank under accession numbers PV186759 and PV186760.

## Introduction

The marine *Roseobacter* group (family *Roseobacteraceae*) represents a phylogenetically diverse lineage within *Alphaproteobacteria*, currently encompassing at least 337 described species across 128 genera [1,2]. As one of the most ecologically significant marine bacterial groups, roseobacters are ubiquitous throughout global marine ecosystems, occupying a wide range of oceanic niches. They typically represent ~20% of coastal and polar ocean bacterioplankton communities and 3–5% of open ocean communities [2,4]. Members of the *Roseobacter* group exhibit remarkable metabolic versatility, mediating key biogeochemical processes through their capacity to degrade a broad spectrum of organic compounds [2,3, 5]. However, their contributions to community abundance and ecological impacts are heterogeneous across different lineages. Metagenomic investigations have shown that several *Roseobacter* lineages, including *Roseobacter* clade-affiliated (RCA), CHAB-I-5, NAC11-7, ChesI-C, SAG-O19 and Clade Hidden and Underappreciated Globally (CHUG), dominate the marine *Roseobacter* communities [2,6,8]. Compared to other known lineages, these dominant lineages exhibit convergent evolutionary adaptations to oligotrophic environments, such as streamlined genomes and enhanced nutrient acquisition systems [2,9, 10]. The unique gene content allowed these lineages to be clustered into a distinct clade, named the pelagic *Roseobacter* cluster (PRC) [7,8].

Roseophages that infect roseobacters play critical roles in regulating community structure, metabolic activity and evolution of roseobacters. Studies on isolated roseophages have revealed significant genomic diversity, providing valuable insights into their diversity and potential ecological functions [11,13]. Currently, a total of 81 roseophages have been isolated and classified into at least 16 distinct phage groups [13,30]. Among these, 32 infect PRC roseobacters, while the remaining phages infect non-PRC roseobacters. The non-PRC roseophages are predominantly composed of those belonging to the *Schitoviridae* [11,14,19, 29], *Cobavirinae* [20,21] and Chi-like groups [22]. The 32 PRC roseophages have been classified into at least 8 distinct phage groups, which mainly belong to the HMO-2011-type and *Autographivirales* [13,24,28]. Similar to the distribution pattern of their hosts, PRC roseophages exhibit higher abundance and broader distributions in marine environments compared to non-PRC roseophages. However, the isolation of PRC roseophages is still limited by the availability of host strains. Most isolated PRC roseophages were obtained using RCA strains as hosts [13,24, 26,28], with only six reported to infect CHAB-I-5, FZCC0037 and CHUG lineages [23,25]. Roseophages infecting the remaining PRC members, such as ChesI-C and NAC11-7 lineages, have not yet been reported.

In this study, seven novel roseophages were isolated from various surface seawater samples using five *Roseobacter* PRC strains as hosts. Phylogenetic and comparative genomic analyses revealed that these roseophages form a distinct taxonomic group, designated CRP-822-type. Additionally, 279 uncultured viral genomes (UViGs) related to these roseophages were identified from the public marine viral database. Phylogenomic analysis established the CRP-822-type phages as a new family comprising at least five subgroups. Finally, their global distribution patterns were investigated through viromic read-mapping analysis.

## Methods

### Host strains isolation

The five *Roseobacter* strains were isolated using a high-throughput dilution-to-extinction method [31]. Four *Roseobacter* strains, FZCC0196, FZCC0197, FZCC0198 and FZCC1911, were isolated from the coastal water of Aoshan Bay, China (36° 36′ N, 121° 9′ E). The strain FZCC0037 was isolated from the coastal water of Pingtan Island, Taiwan Strait, China (25° 15′ N, 119° 28′ E). All strains were purified at least three times and grown in the dark at 26 °C in natural seawater-based medium supplemented with 1 mM NH_4_Cl, 100 µM KH_2_PO_4_, 1 µM FeCl_3_ and mixed carbon sources [32]. Bacterial density was determined using a Guava EasyCyte flow cytometer (Millipore, Guava Technologies) after staining with SYBR Green I (Invitrogen). The 16S rRNA genes of the five roseobacters were amplified using the primers 27F (5′-AGAGTTTGATCMTGGCTCAG-3′) and 1492R (5′-TACGGTTACCTTGTTACGACTT-3′) [33], and the 16S rRNA gene sequences were obtained through Sanger sequencing.

### Phage isolation and purification

The five *Roseobacter* strains were used to isolate roseophages from diverse seawater samples collected at several coastal stations (Table 1) [23,24]. Initially, the seawater samples were filtered through 0.1-μm-pore-size sterile syringe filters and stored in the dark at 4 °C until use. Subsequently, the filtered seawater was added to exponentially growing host cultures, and cell growth was monitored using a Guava EasyCyte flow cytometer. Upon detecting a decrease in host cell density, the release of phage particles was confirmed through epifluorescence microscopy. The dilution-to-extinction method was performed to purify the phages at least three times.

**Table 1.** General characteristics of the seven isolated roseophages

Genome	Host	Station	Latitude	Longitude	Collection date	Length (bp)	G+C mol%	No. of ORFs	GenBank ID
CRP-811	FZCC0198	Yantai	37° 28′ N	121°28' E	April 2021	32,559	52.8	49	PV211010.1
CRP-821	FZCC0197	Taizhou	28° 28′ N	121°40' E	February 2022	30,260	52.42	43	PV211011.1
CRP-822	FZCC0197	Yantai	37° 27′ N	121°42' E	December 2021	30,260	52.42	43	PV211012.1
CRP-823	FZCC0197	Yantai	37° 28′ N	121°28' E	April 2021	29,331	52.1	44	PV211013.1
CRP-831	FZCC0196	Yantai	37° 28′ N	121°28' E	April 2021	30,973	51.94	44	PV211014.1
CRP-406	FZCC0037	Taizhou	28° 28′ N	121°40' E	February 2022	29,144	42.8	42	PV211008.1
CRP-701	FZCC1911	Taizhou	28° 28′ N	121°40' E	February 2022	29,189	47.88	42	PV211009.1

### DNA preparation and sequencing

Each phage lysate was filtered through 0.1-μm-pore-size filters and concentrated to ~1 ml using Amicon Ultra centrifugal filters (30 kDa, Millipore). The filtered lysate was then concentrated using ultracentrifugation (Beckman Coulter, USA) at 40,000×***g*** for 2 h. Subsequently, the DNA of roseophages was extracted using the DNeasy Blood and Tissue kit (QIAGEN). The phage DNA library was sequenced using Illumina paired-end HiSeq 2,500 sequencing with 2×150 bp by Novogene (Beijing, China). FASTP v.0.23.2 with default settings was used to trim and remove adaptors and low-quality reads from the raw data [34]. The phage genomes were assembled using MEGAHIT v.1.2.9 with default settings [35].

### Transmission electron microscopic analysis

The morphology of purified phages was observed using transmission electron microscopy (TEM). Briefly, each phage lysate was filtered using a 0.1-μm-pore-size filter. The filtered phage lysate was concentrated using Amicon Ultra centrifugal filters and ultracentrifugation at 40,000×***g*** for 2 h. The concentrated phages were deposited onto a copper TEM grid and air-dried. The grid was stained with 2% uranyl acetate for 2 min and observed using a Hitachi TEM at 80 kV.

### The growth curves of host and phage

For the growth curve analysis of the host, purified CRP-822 was added to the exponentially growing cultures of FZCC0197 with a phage-to-bacterial ratio of ~3:1. For control samples, an equal volume of culture medium was added to the FZCC0197 cultures. The cell density of FZCC0197 was monitored using a Guava EasyCyte flow cytometer. For the growth curve of CRP-822, exponentially growing cultures of FZCC0197 were exposed to CRP-822 at a phage-to-bacterial ratio of ~1 : 10. The relative abundance of phages was quantified using quantitative PCR (qPCR). qPCR primers were designed to target the DNA helicase sequence using the NCBI (National Center for Biotechnology Information) Primer-blast service. The qPCR was conducted using the Taq Pro Universal SYBR qPCR Master Mix kit with the following cycling conditions: initial denaturation at 95 °C for 3 min, followed by 40 cycles at 95 °C for 5 s and 60 °C for 15 s. Samples were taken for cell counting and qPCR analysis every 30 min during the experiment. All experiments were performed in triplicate.

### Metagenomic retrieval of CRP-822-type UViGs

To extend the diversity of CRP-822-type phages in marine environments, we retrieved UViGs related to the seven roseophages from IMG/VR v.4 [36] and Delaware Bay and Chesapeake Bay Viromes [37]. Based on the amino acid sequences of the hallmark genes, including DNA helicase, capsid and terminase large subunit (TerL), we created profile hidden Markov models (HMM) using the hmmbuild command of HMMER v.3.3.2 [38]. These HMM profiles were used to retrieve UViGs from databases using the hmmsearch program of HMMER (*e*-value ≤10^−3^; bit score ≥50) [38]. Only the UViGs that contain all three hallmark genes were considered. The CheckV v.1.0.1 [39] and cd-hit v.4.8.1 [40] were used to eliminate low-quality (<80% completeness) and redundant sequences (>95% identity). Finally, 279 UViGs were retained for further analysis.

### Genome annotation and comparison

The ORFs of the phage genomes were predicted using the Prodigal v. 2.6.3 [41] and GeneMarkS online server [42]. The functional annotation of the translated ORFs was performed using blastp against the NCBI non-redundant and the NCBI RefSeq databases (> 25% amino acid sequence identity and *e*-value <10^−3^). The Pfam database was used to identify protein families and conserved domains [43]. Orthologous genes of the CRP-822-type phages were identified and clustered using OrthoFinder [44]. Representative UViGs were selected from each subgroup based on core gene phylogeny and genomic completeness. These representative genomes were then compared pairwise using blastp and visualized using Easyfig v.2.2.2 [45]. The average amino acid identity (AAI) values, amino acid usage and codon usage were calculated using the CompareM (https://github.com/donovan-h-parks/CompareM).

### Phylogenetic and network analyses

To clarify the phylogenetic position of the five *Roseobacter* strains, we constructed a phylogenetic tree based on the 16S rRNA gene sequences. The tree was constructed using IQ-TREE with the ModelFinder assigning the best substitution model and 1,000 ultrafast bootstrap [46].

The vConTACT2 [47] and PhaGCN2 [48] with default parameters were used to evaluate the taxonomy level of CRP-822-type phages. The networks created by vConTACT2 and PhaGCN2 were visualized using Cytoscape v.3.5.1 with an edge-weighted spring-embedded layout. Meanwhile, the whole-genome phylogenetic tree was constructed using ViPTree and VirClust (https://www.genome.jp/viptree/ and http://virclust.icbm.de) [49,50]. The phylogenetic tree based on the amino acid sequences of conserved genes of CRP-822-type phages, including DNA helicase, capsid and TerL, was generated using the maximum likelihood algorithm in IQ-TREE [46]. Additionally, to clarify the evolutionary relationships of CRP-822-type phages, the amino acid sequences of five core genes, including those encoding DNA helicase, capsid, portal, tail length tape measure protein and TerL, were sequentially trimmed using trimAl [51] and aligned using MAFFT [52]. The trimmed alignments were concatenated using a custom script. The core gene phylogenetic tree of CRP-822-type phages was constructed using IQ-TREE [46]. All the resulting phylogenetic trees were visualized with iTOL v.5 [53].

### Host prediction

The potential hosts of CRP-822-type UViGs were predicted using the iPHoP framework with default settings [54]. The iPHoP integrated several host prediction tools, including RaFAH and WIsH. A confidence score cutoff of 90% was applied for the final predictions [55,56].

### Ecological distribution

The global distribution of CRP-822-type phages was assessed by viromic read-mapping analysis. This analysis utilized a comprehensive dataset of 220 marine viromic databases, which included the Global Ocean Viromes 2.0 [57], Mariana Trench Virome [58], Pearl River Estuary virome [59], Delaware Bay and Chesapeake Bay viromes [37] and others [60,63]. Viromic reads were mapped to the non-redundant set of CRP-822-type phage genomes using CoverM v.0.6.1 (>95 % nucleotide identity, ≥50 bp aligned length, ≥80 % read coverage) (https://github.com/wwood/CoverM). The relative abundance was normalized by reads per kilobase per million reads (RPKM). Differences in the relative abundance of CRP-822-type phages were evaluated using the Mann–Whitney *U* test in R v.4.3.2. Correlations between phage relative abundance and environmental factors was calculated in R v.4.3.2. All statistical results were plotted using the package ggplot2 in R.

## Results and discussion

### General biology of the seven isolated phages

Five *Roseobacter* strains were utilized as hosts to isolate roseophages. Among these, three strains (FZCC0196, FZCC0197, FZCC0198) shared 99% 16S rRNA sequence identity with the previously reported CHUG strain HKCCA1288. FZCC1911 exhibited 99.8% sequence identity with the ChesI-C strain HIMB11. The remaining strain, FZCC0037, has been previously described [24] and was identified as a new PRC member (Fig. S1A, available in the online Supplementary Material). The 16S rRNA gene phylogenetic tree further confirmed their taxonomic classification (Fig. S1B).

**Fig. 1.**
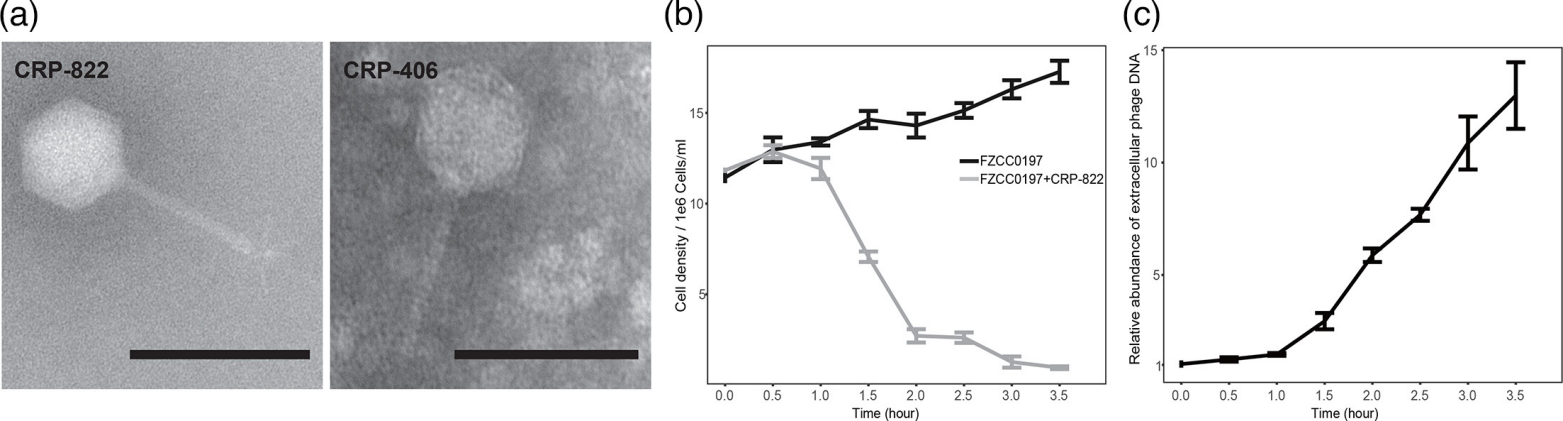
General biological characteristics of roseophages CRP-822 and CRP-406. (**a**) Transmission electron microscopy images of CRP-822 and CRP-406. Scale bar, 100 nm. (**b**) Growth curves of the host FZCC0197 with and without CRP-822 infection. (**c**) Growth curve of the CRP-822 in exponential cultures of FZCC0197. The *Y*-axis shows the relative abundance of extracellular phage DNA, which was calculated by normalizing the copy number of the DNA helicase gene at each time point to that at the initial time point (T0).

A total of seven roseophages were isolated from coastal waters (Table 1). CRP-821, CRP-822 and CRP-823 infect CHUG strain FZCC0197, while CRP-811 and CRP-831 infect CHUG strains FZCC0196 and FZCC0198, respectively. Additionally, CRP-701 and CRP-406 infect the ChesI-C strain FZCC1911 and FZCC0037, respectively. After three rounds of purification procedures, CRP-822 and CRP-406 were successfully purified. However, the other five roseophages could not be purified due to contamination with other types of phages. TEM analysis showed that CRP-822 has an icosahedral capsid with an average diameter of 63.88±1.00 nm, a long non-contractile tail of 89.22±1.47 nm and tail fibres of 19.15±0.60 nm (Fig. 1a). CRP-406 exhibits a similar morphology, having an icosahedral capsid with a diameter of 59.20±0.63 nm and a non-contractile tail of 81.20±1.59 nm (Fig. 1a). These morphological features are characteristic of siphovirus morphology. Growth curve analysis demonstrated that host cell density decreased at 1 h post-infection with CRP-822, coinciding with an increase in phage DNA at the same time (Fig. 1b, c).

### CRP-822-type phages represent a new phage family

These isolated roseophages exhibited genome sizes ranging from 29.14 to 32.56 kb and G+C contents ranging from 42.80 to 52.80%. They were predicted to contain 42–47 ORFs, of which ~45% have been annotated with putative biological functions (Table S1). No tRNA genes were identified in these roseophages.

**Fig. 2.**
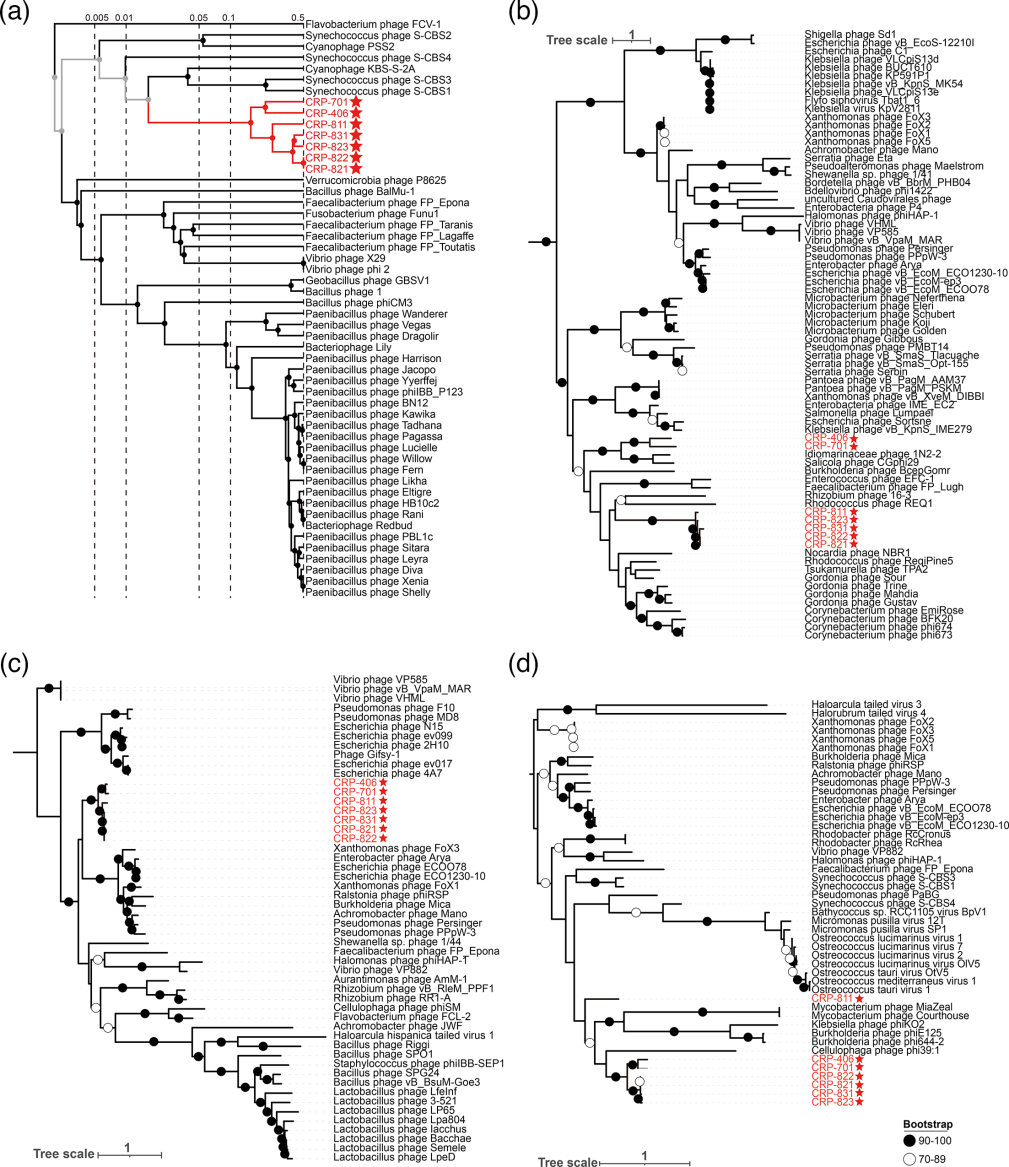
Phylogenetic analyses of the seven isolated roseophages. (**a**) Genome-wide proteomic tree constructed using VipTree based on the genomes of seven isolated roseophages and all known dsDNA phages. For clarity, only the branches containing the seven roseophages and their related phages are displayed. Phylogenetic trees constructed using sequences of the DNA helicase (**b**), terminase large subunit (TerL) (**c**) and capsid (**d**).

Comparative genomic analysis revealed that the seven roseophages shared few genes with other known roseophages, which is consistent with their distinct position in the whole-genome protein phylogenetic tree (Fig. S2). To elucidate their evolutionary relationships, we constructed a broader whole-genome protein phylogenetic analysis, which included these seven roseophages and other known phages, This analysis showed that the seven roseophages formed a novel branch, clustering near *Synechococcus* phages S-CBS1 and S-CBS3 (Fig. 2a) [64]. However, they shared only 5–7 genes with these cyanophages. AAI analysis demonstrated that seven roseophages shared an average of 60% AAI with each other (Table S2). Collectively, these results suggest that the seven roseophages constitute a novel phage group, which we tentatively designated the CRP-822-type.

Furthermore, three core genes, including the DNA helicase, TerL and capsid genes, were used to elucidate their evolutionary relationships with other known phages. The DNA helicase phylogeny showed that the five CHUG phages grouped into a unique clade, whereas CRP-406 and CRP-701 formed another branch adjacent to the *Idiomarinaceae* phage 1N2-2 and *Salicola* phage CGphi29 (Fig. 2b). This suggests that the DNA helicase genes of the five CHUG phages exhibit a distant evolutionary relationship with those in CRP-406 and CRP-701 and may have independently evolved to adapt to their distinct hosts’ nucleic acid synthesis and metabolic pathways. TerL plays a pivotal role in recognizing, binding and translocating phage genome into the capsid and also serves as an endonuclease that cuts the concatemeric DNA into single genomic molecules [65,66]. Phylogenetic analysis of the TerL gene indicated that all seven roseophages constituted a unique branch (Fig. 2c). Moreover, the phylogenetic tree based on capsid genes revealed that CRP-811 formed a separate branch that was divergent from the other six roseophages (Fig. 2d). This suggests that the capsid gene of CRP-811 may have experienced a unique evolutionary trajectory, despite the overall conservation among capsid genes. Collectively, although variation was observed in their DNA helicase and capsid proteins, phylogenetic and AAI analyses supported the classification of the seven roseophages within a novel phage group.

**Fig. 3.**
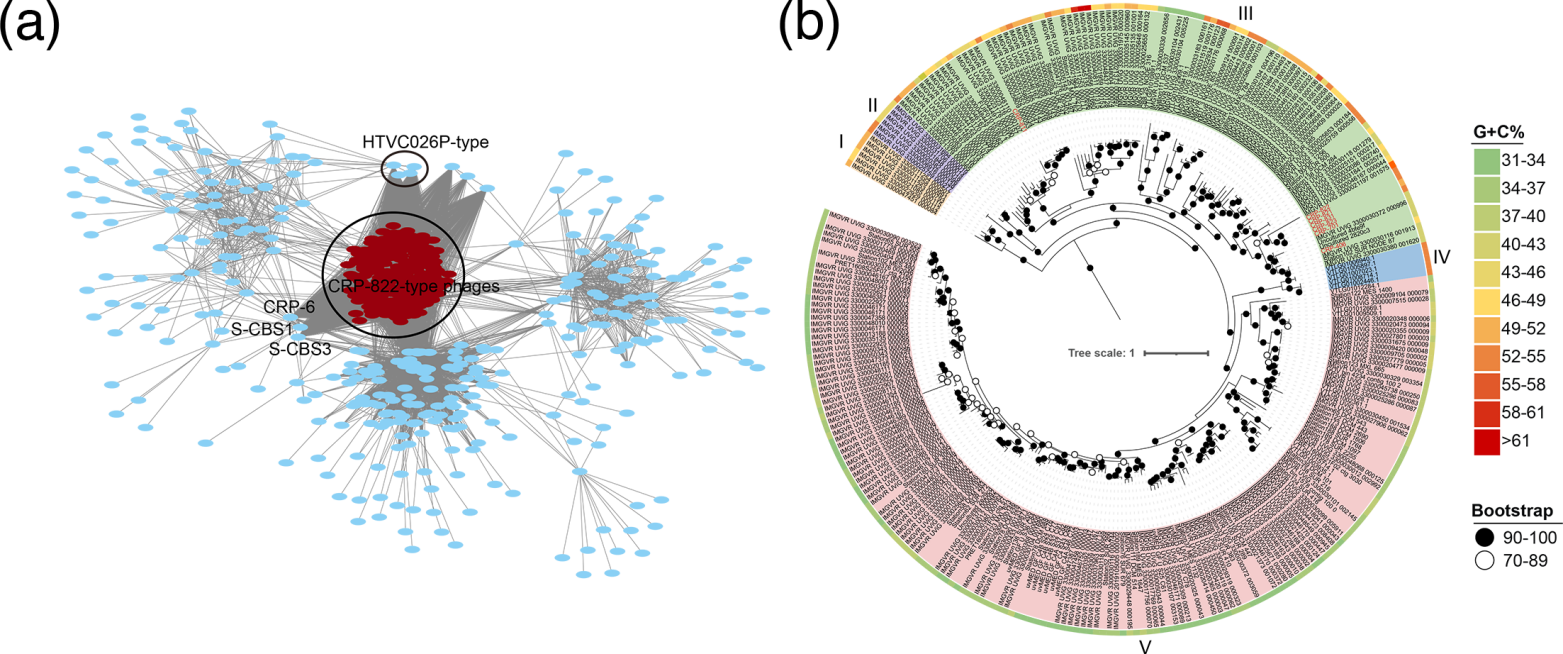
Evidence supporting the taxonomy of CRP-822-type phages. (**a**) Gene content-based viral network of CRP-822-type roseophages, related UViGs and other known viruses from the NCBI-RefSeq database, constructed with vConTACT2. For clarity, only the known viruses related to CRP-822-type phages are presented. CRP-822-type phages are coloured in red. (**b**) Phylogenetic tree of CRP-822-type phages based on the concatenated amino acid sequences of five core genes. The five subgroups and G+C content are indicated by different colours. The seven isolated roseophages are marked in red.

To further investigate the diversity of this novel phage group, a total of 279 non-redundant UViGs closely related to the 7 roseophages were retrieved from virus genome databases. The genome size of the retrieved UViGs varied from 27.36 to 39.33 kb with at least 80% genome completeness, and their G+C contents ranged from 31.36 to 61.51% (Table S3). VipTree analysis showed that the seven roseophages and these UViGs formed a distinct clade, separate from all currently classified phage groups (Fig. S3A). This classification was further supported by VirClust, which confirmed these UViGs as members of the CRP-822-type group (Fig. S3B). Consistent with these findings, both vConTACT2 and PhaGCN2.0 analyses also suggest that these genomes formed a novel viral cluster at the family level (Figs 3a and S3C). Furthermore, phylogenomic analysis based on the five core genes revealed that the CRP-822-type group contained at least five well-supported subgroups (Fig. 3b). Among these, subgroups III and V contained the largest numbers of members, with 99 and 168 members, respectively. All seven roseophages were classified into subgroup III. Collectively, these findings provide strong evidence that the CRP-822-type phages constitute a novel phage family, comprising at least five distinct subgroups.

### Conserved genomic architecture and variations in CRP-822-type phages

**Fig. 4.**
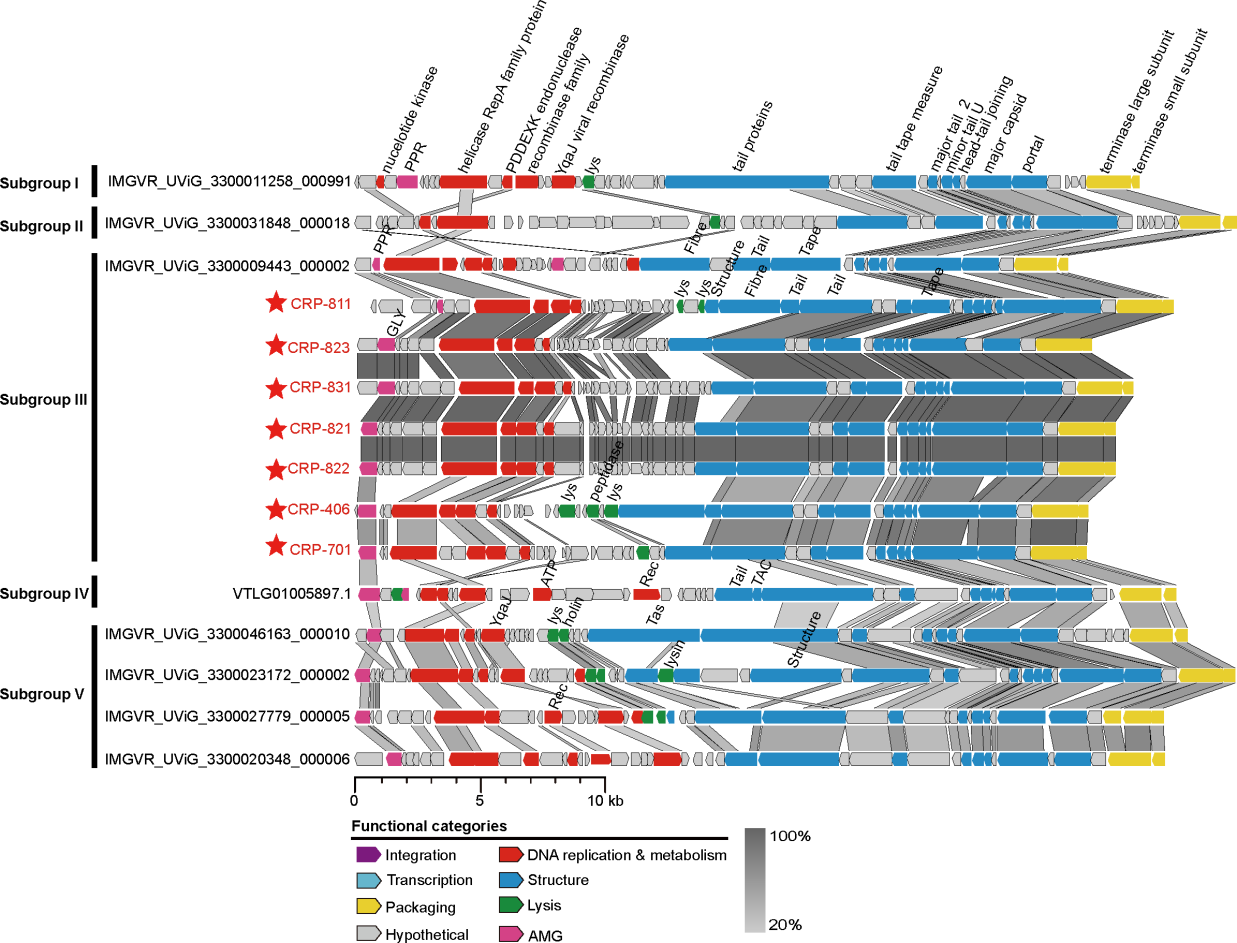
Genomic comparison of CRP-822-type phages from the five major subgroups. Seven isolated roseophages are indicated by red asterisks. Representative UViGs with high genomic completeness were selected from each subgroup based on core gene phylogeny. Predicted ORFs are represented by arrows, with the left or right arrow points indicating the direction of their transcription. ORFs annotated with known functions are marked using distinct colours according to their functions. PPR, phosphoadenosine phosphosulphate reductase; GLY, glycosyltransferase; lys, lysozyme; Tape, tape measure protein; Rec, recombinase; TAC, tail assembly chaperone; YqaJ, YqaJ viral recombinase; Tail, tail protein; Fibre, fibre protein; Structure, structure protein.

Genomic analysis revealed that all CRP-822-type phages share a conserved genomic architecture (Fig. 4). A total of 746 orthologous protein groups (>2 members) were identified in their genomes, of which 293 could be assigned putative biological functions. Twenty genes were designated as core genes, including those encoding the capsid, DNA helicase, TerL, portal protein and terminase small subunit (TerS) (Table S4). The integrase gene was absent in almost all CRP-822-type phages, suggesting that they may follow a strict lytic life cycle.

In the DNA replication and metabolism module, the gene encoding DNA polymerase, which is primarily responsible for DNA replication, was present in some members of subgroups I, III and V, but was absent in subgroups II and IV (Fig S4). This suggests that members of these subgroups rely on the host replication machinery for propagation. Except for subgroup IV, the gene encoding DNA methyltransferase was identified in most members of other subgroups (Fig S4). This gene is involved in DNA methylation as a strategy to counteract host defence mechanisms [67]. The gene encoding the single-stranded DNA-binding protein (SSB) was mainly identified in subgroups II and III. SSB is widely distributed across all kingdoms of life and among DNA viruses, where it plays a vital role in protecting single-stranded DNA intermediates during replication, repair and recombination [68]. These findings underscore the diverse mechanisms employed by CRP-822-type phages for DNA replication and metabolism and highlight their adaptive strategies for interacting with and exploiting host cells.

The packaging and structural module of CRP-822-type phages showed a high degree of conservation, with 13 genes consistently present across these phages. These included genes encoding the TerS, TerL, major capsid protein, portal protein and head-tail-joining protein (Figs 4 and S4), suggesting that the CRP-822-type phages share a similar morphogenesis and packaging mechanism. In contrast, the tail assembly chaperone, fibrinogen protein and distal tail protein were restricted to specific subgroups. Tail-related genes can be subdivided into numerous orthologous groups (Table S4). These observations imply that the tail-related genes of CRP-822-type phages may have undergone rapid evolution to enhance the host recognition and infection ability, thereby expanding their host range [69,71]. Additionally, five genes associated with host cell lysis and the release of progeny virions were identified. Among these, genes encoding holin and endolysin were present in most CRP-822-type phages (Figs 4 and S4). These genes can degrade the peptidoglycan of the bacterial host, facilitating the release of progeny virions [72,73].

Auxiliary metabolic genes (AMGs) are presumed to originate from the host and play roles in the regulation of host metabolism during infection. A total of five AMGs were identified in the CRP-822-type phages (Figs 4 and S4). The dCMP deaminase gene, which can hydrolyse dCMP into dUMP for thymidylate synthase and is regulated by the ratio of dCTP to dTTP [74], was found only in a few members of subgroups III and V (Figs 4 and S4). The gene encoding triphosphate pyrophosphohydrolase, which is likely involved in histidine biosynthesis and the hydrolysis of dUTP into dUMP, was found exclusively in subgroups III and V (Figs 4 and S4) [75]. The presence of these two genes, which are involved in *de novo* DNA synthesis, provides CRP-822-type phages with advantages during infection. They enhance the phage’s ability to effectively utilize the host resources and machinery for replication. The gene encoding phosphoadenosine phosphosulphate reductase (PAPS), which catalyses the reduction of PAPS to phosphoadenosine phosphate, was found in a subset of members of subgroups I, III and V (Fig S4). This enzyme likely plays a role in sulphur metabolism, potentially contributing to processes such as sulphur assimilation, which are important for various cellular functions [76,77]. Additionally, two genes encoding glucosyltransferase and phosphate adenyltransferase were identified in only two CRP-822-type phages (Fig S4). However, the specific biological functions of these two genes in phages remain unclear.

### Differential genomic features of CRP-822-type phage subgroups

**Fig. 5.**
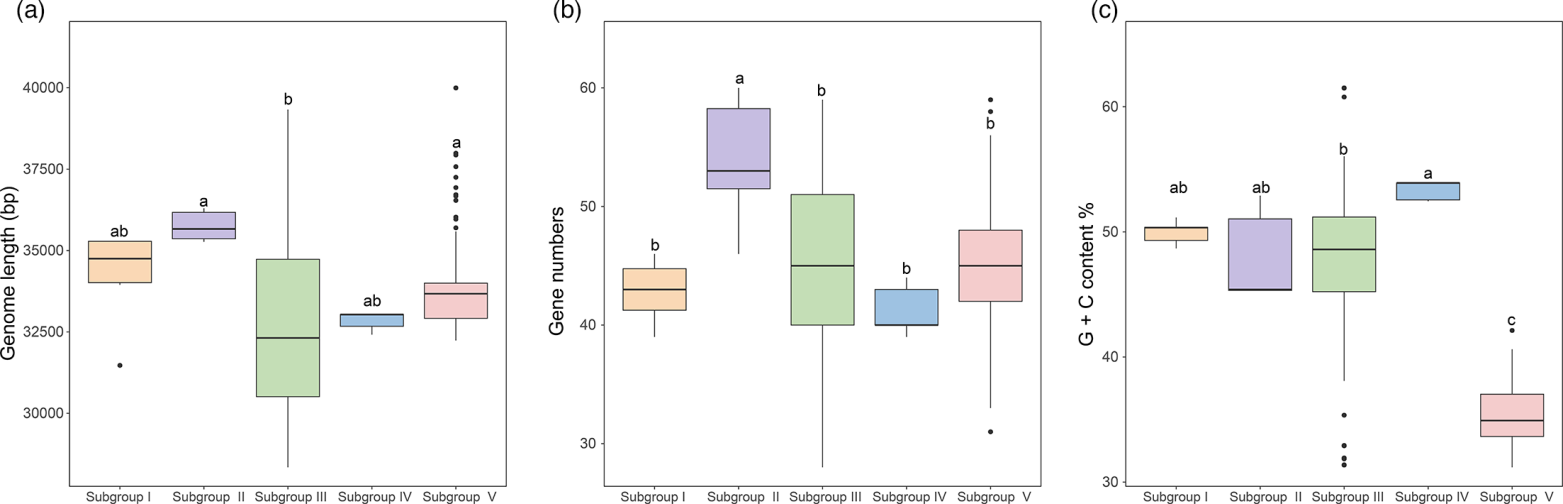
Comparison of genomic features across the five CRP-822-type subgroups. (**a**) Genomic length, (**b**) number of genes and (**c**) G+C content. The significance of pairwise comparisons is indicated by different letters above the boxes (*P*<0.05).

The CRP-822-type phages exhibit significant genomic variation across the five subgroups. Subgroup II possesses the largest genome (35.75±0.46 kb) and the greatest number of ORFs (53.8±5.42) (Fig. 5a, b). Subgroup III and V show considerable variation in genome sizes, ranging from 28,339-39,333 bp and 32,234-39,996 bp, respectively (Fig. 5a). Among the five subgroups, subgroup V has the lowest G+C content (35.4±2.25%) (Fig. 5c). Notably, some members of subgroup III, which formed a separate branch, also have low G+C content (32.53±1.30%) (Fig. 3b). Moreover, variations in amino acid and codon usage were also observed among different subgroups. For example, low G+C subgroup V members exhibit a different amino acid usage preference compared to other four subgroups. Specifically, subgroup V members show increased usage of asparagine, isoleucine, lysine, tyrosine and serine, while showing decreased usage of alanine, arginine, glycine, proline and valine (Fig. S5A). Additionally, subgroup V members and the low G+C subgroup III members tend to favor A/T-rich codons (Fig. S5B). Generally, phages maintain a similar G+C content, amino acid usage and codon usage with their hosts to adapt to the host’s DNA replication mechanisms and gene expression patterns [78]. Therefore, these observed variations may imply that CRP-822-type phages can infect diverse bacterial hosts. Host prediction analysis suggested that CRP-822-type phages may infect different bacterial groups, including SAR86, SAR11, KI89A and *Flavobacteriales* (Table S5). The SAR86 clade, characterized by streamlined genomes and low G+C content, is one of the most abundant yet uncultivated bacterial groups in surface ocean microbial assemblages [79]. It is the predicted host for most members of subgroup V (Table S5). The potential host of subgroup IV was predicted to be the KI89A clade (HTCC2089) (Table S5) [80]. Many high-G+C members of subgroup III were predicted to infect *Roseobacteracea*, while those with low G+C content were predicted to infect SAR11 and *Roseobacter* NAC11-7 lineages (Table S5).

### Ecological distribution of CRP-822-type phages in the ocean

**Fig. 6.**
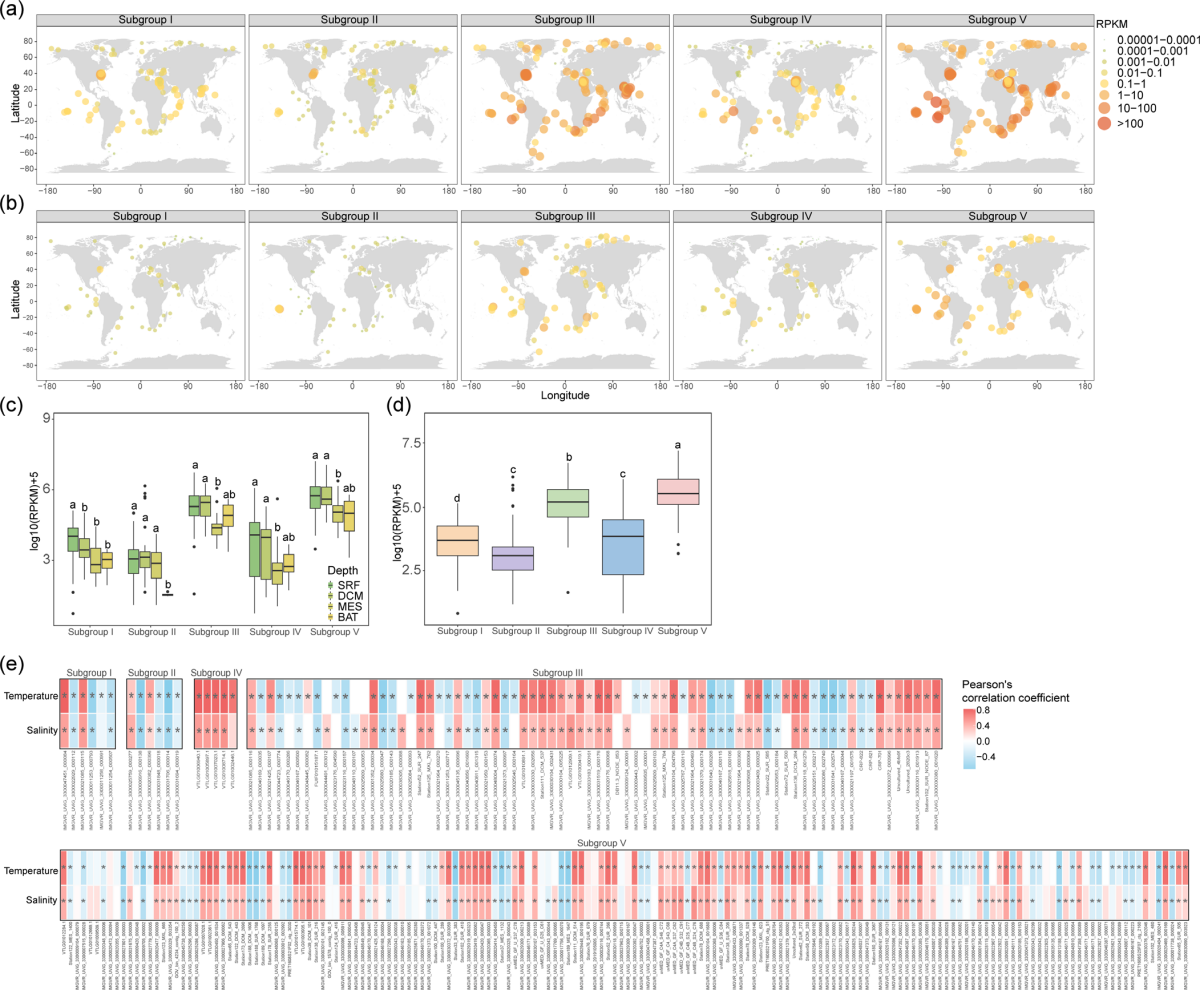
Global distribution of CRP-822-type phages and their ecological correlation with environmental factors. Global maps showing the RPKM values of CRP-822-type phages in (**a**) surface and (**b**) DCM viromes. The size and colour of the dots represent the RPKM values. (**c**) Comparison of RPKM values of different subgroups at different depths. (**d**) Comparison of RPKM values among different subgroups. The different letters above the boxes indicate significance with a *P*-value<0.05. (**e**) Correlation between the relative abundance of different subgroups and temperature and salinity from the TARA Ocean metagenomic. Red and blue indicate positive and negative correlations, respectively. Asterisks indicate significance with a *P*-value <0.05.

The biogeographic distribution of CRP-822-type phages was investigated using metagenomic read-mapping analysis of 220 marine viromic datasets. All five CRP-822-type subgroups were detected from polar to trade biomes and were primarily detected in surface waters and the deep chlorophyll maximum (DCM) layer (Fig. 6a–c). Among the 286 CRP-822-type phages, 246 (86.0%) exhibited broad distribution, being detected in over 50 marine viromes (Fig. S6 and Table S6). The seven new roseophages in subgroup III, despite being isolated from coastal surface waters, also displayed widespread distributions (Fig. S6 and Table S6). Among them, CRP-701 was the most prevalent, being detected in 138 viromic datasets and showing a prevalent distribution in trade and westerlies biomes (Fig. S6 and Table S6). This distribution pattern aligns with the distribution of its host, ChesI-C, in these environments [7]. The five CHUG phages (CRP-811, CRP-821, CRP-822, CRP-823, and CRP-831) were more frequently detected in the polar and estuarine biomes (Fig. S6), whereas CRP-406 was largely restricted to estuaries (Fig. S6).

CRP-822-type phages exhibited overall higher relative abundance than previously reported HMO-2011-type RCA phages CRP-1 and CRP-2, as well as *Autographivirales* SAR11 phage HTVC109P (Fig. S6). This indicates that CRP-822-type phages represent an abundant phage group in the ocean. Notably, subgroups III and V had significantly higher RPKM values than other subgroups (*P*<0.05, Fig. 6a, c and d), and their members occupied distinct niches (Fig. S6). Specifically, approximately half of the members in subgroups III and V thrived in trade and westerlies biomes (Fig. S6) and correlated positively with temperature and salinity (*P*<0.05, Fig. 6e). In contrast, some members in subgroups III and V displayed opposite patterns with higher abundance in polar biomes and negative correlations with temperature(Figs 6e and S6). Divergent biogeographic patterns were also observed in subgroups I and II. All subgroup IV members were more abundant in trade, westerlies and coastal biomes but were scarce in polar biomes and estuaries (Fig. S6). Conversely, some members of the other four subgroups were prevalent in estuaries. Correlation analyses revealed that the relative abundance of subgroup IV was strongly associated with temperature and salinity (*P*<0.05, Fig. 6e). Overall, the divergent biogeographic patterns of CRP-822-type phages suggest that niche differentiation within and among subgroups is driven synergistically by environmental conditions and host distribution dynamics.

## Conclusion

This study reports the isolation of seven roseophages infecting three PRC lineages and proposes a novel family-level phage group designated the CRP-822-type. This group comprises at least five subgroups with distinct genomic and ecological features. Global metagenomic read-mapping revealed that CRP-822-type phages are abundant and widely distributed in the global ocean, indicating their adaptations to diverse marine environments. Host prediction suggests that some of these phages may infect diverse bacterial lineages, including SAR11, SAR86, KI89A and *Flavobacteriales*, highlighting their key roles in regulating these dominant marine heterotrophic bacteria. Our findings expand knowledge of marine roseophage diversity and evolution and discovered a novel and ecologically significant phage group. Nevertheless, the full extent of host diversity remains unresolved due to challenges in culturing marine bacteria. Future research employing alternative culturing approaches or single-cell genomics will be crucial for identifying and verifying the hosts of CRP-822-type phages.

## Supplementary material

10.1099/mgen.0.001568 Uncited Supplementary Material 1.

10.1099/mgen.0.001568 Uncited Supplementary Material 2.

## Abbreviations

AAI: average amino acid identity
AMGs: auxiliary metabolic genes
CHUG: Clade Hidden and Underappreciated Globally
DCM: deep chlorophyll maximum
HMM: hidden Markov models
PAPS: phosphoadenosine phosphosulphate reductase
PRC: pelagic *Roseobacter* cluster
qPCR: quantitative PCR
RCA: *Roseobacter* clade-affiliated
RPKM: reads per kilobase per million reads
TEM: transmission electron microscopy
UViGs: uncultured viral genomes
