# Supplementary material for: Novel roseophages provide insights into a genetically and ecologically diverse phage family
Source: Microb Genom. 2025 Nov 19;11(11):001568. doi: 10.1099/mgen.0.001568 (PMC12629253; doi:10.1099/mgen.0.001568)
Supplement: Uncited Supplementary Material 1. [file mgen-11-01568-s001.pdf]

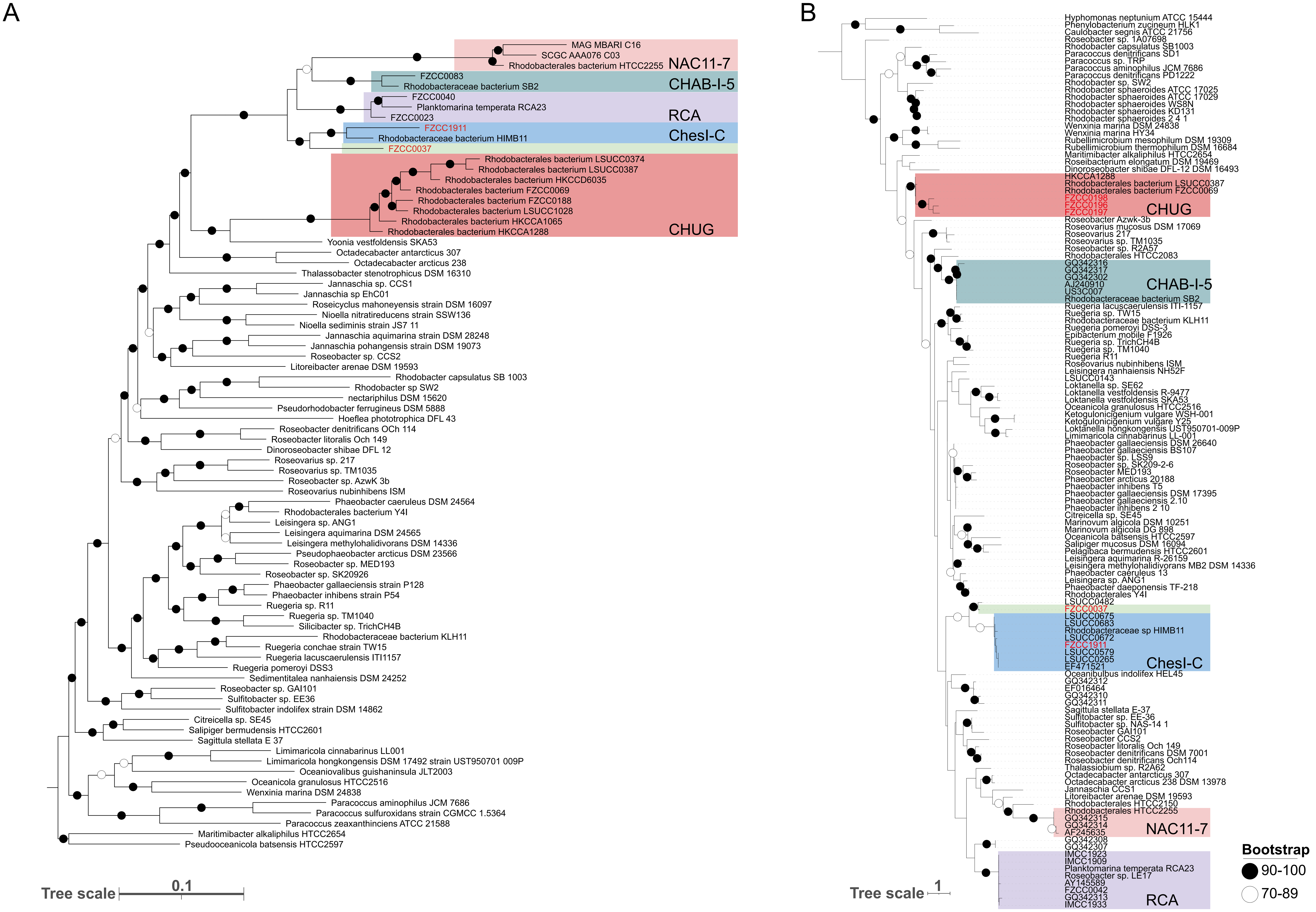

Figure S1. Phylogenetic tree of Roseobacter. (A) Dendrogram of the Roseobacter genomes based on the presence/absence pattern of orthologous gene families. (B) Phylogenetic tree based on the 16S rRNA gene sequences of Roseobacter. The host strains FZCC0196, FZCC0197, FZCC0198, FZCC0037, and FZCC1911, are labeled in red. Circles in the phylogenic tree indicate bootstrap values.

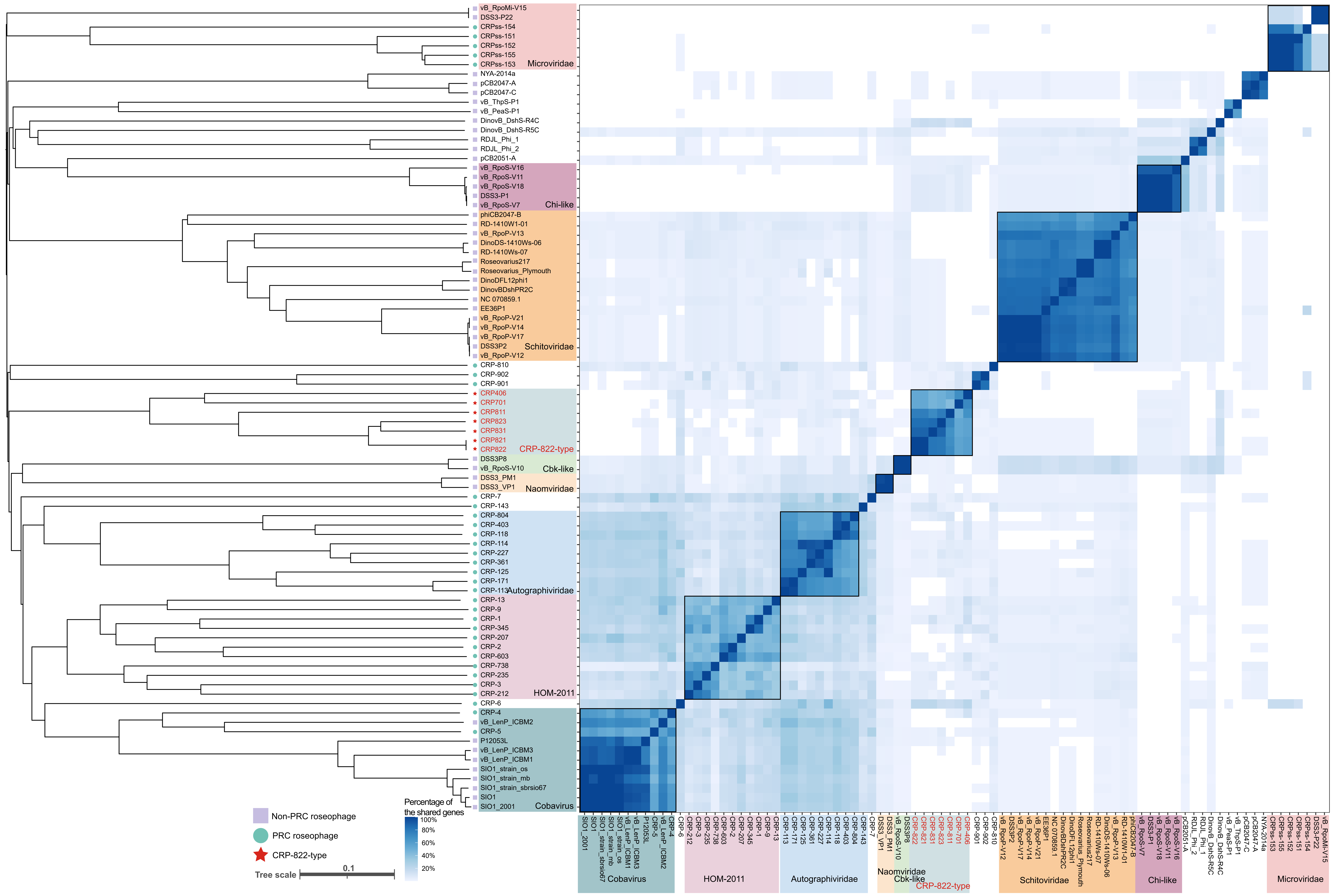

Figure S2. A genome-wide proteomic tree and heatmap of the percentage of shared genes. Different colors represent different phage groups

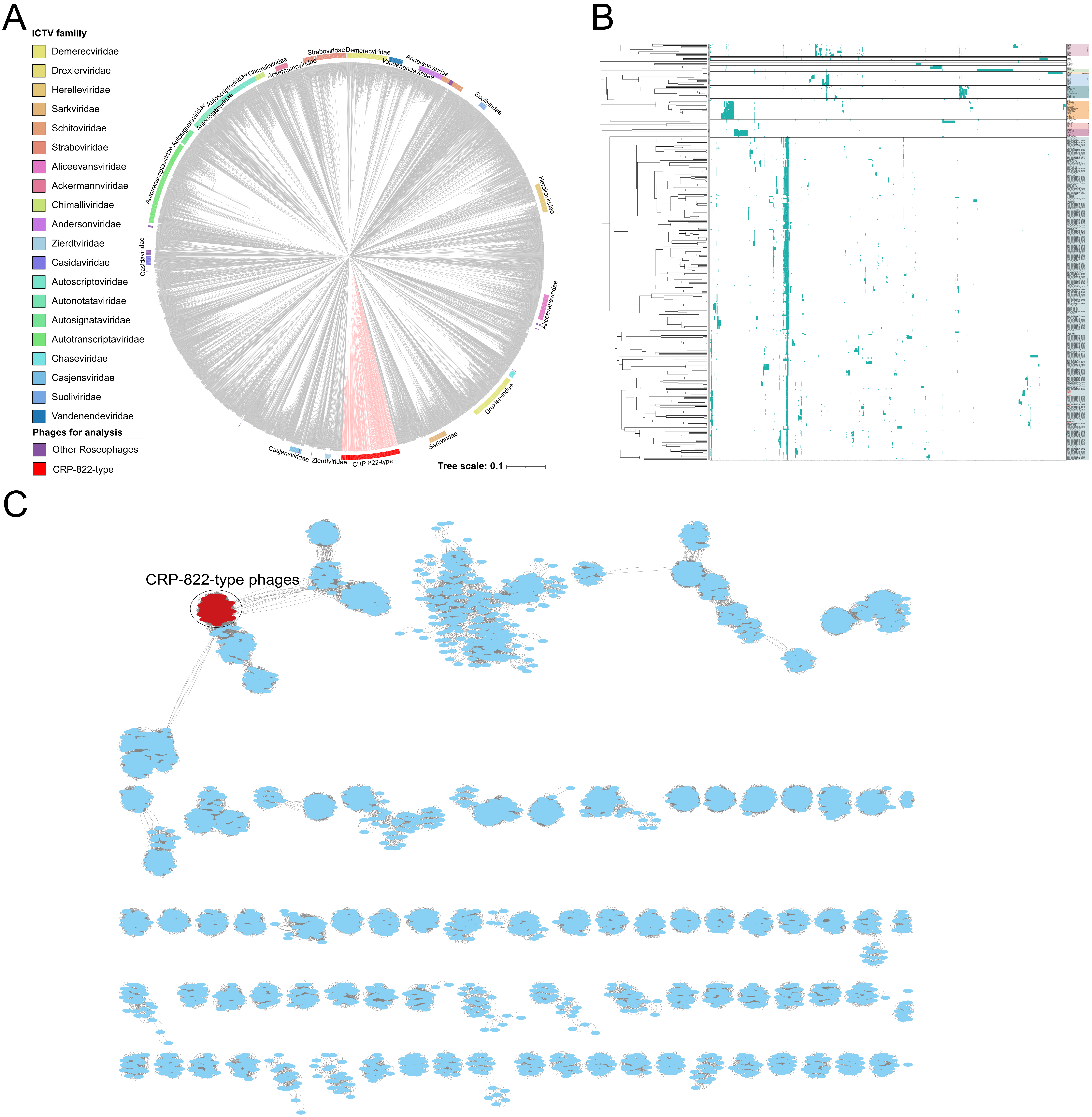

Figure S3. The CRP-822-type phages constitute a new phage family. (A) Genome-wide proteomic tree constructed using VipTree. (B) The phylogenetic tree based on protein clustering using VirCluster. (C) Network of viruses genomic clustering for CRP-822-type phages and RefSeq database using PhaGCN2.

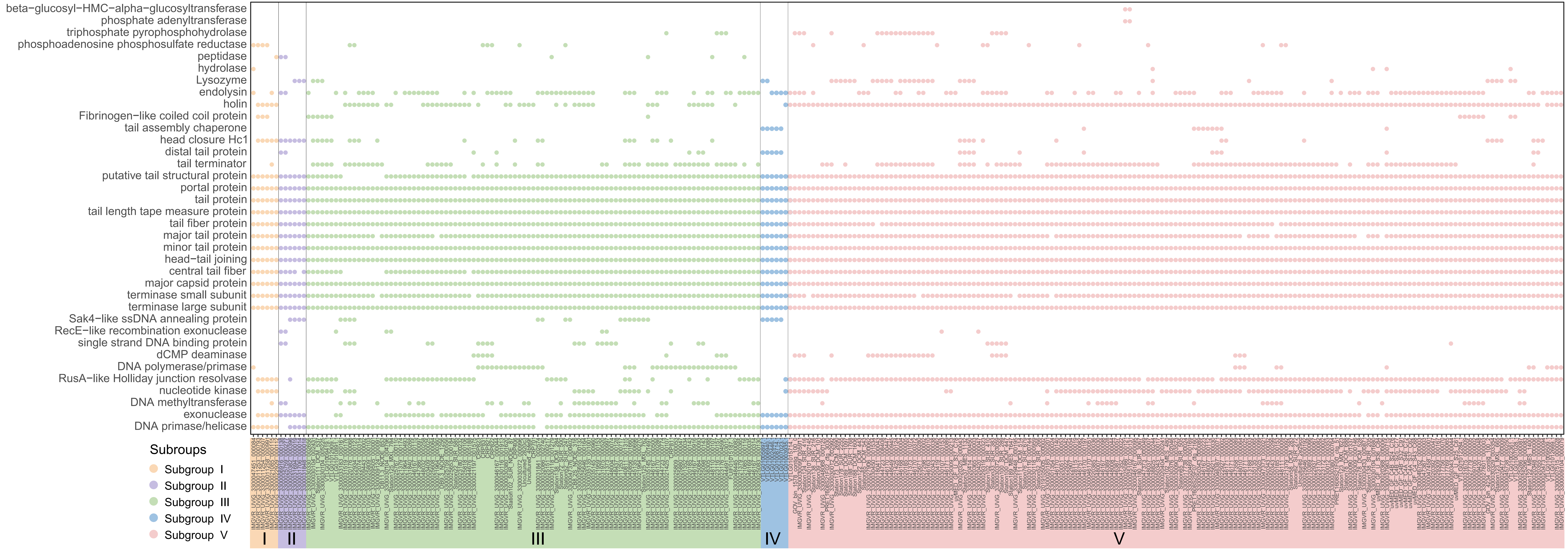

Figure S4. Distribution and functional classification of orthologous groups across CRP-822-type genomes. Different colors represent different subgroups.

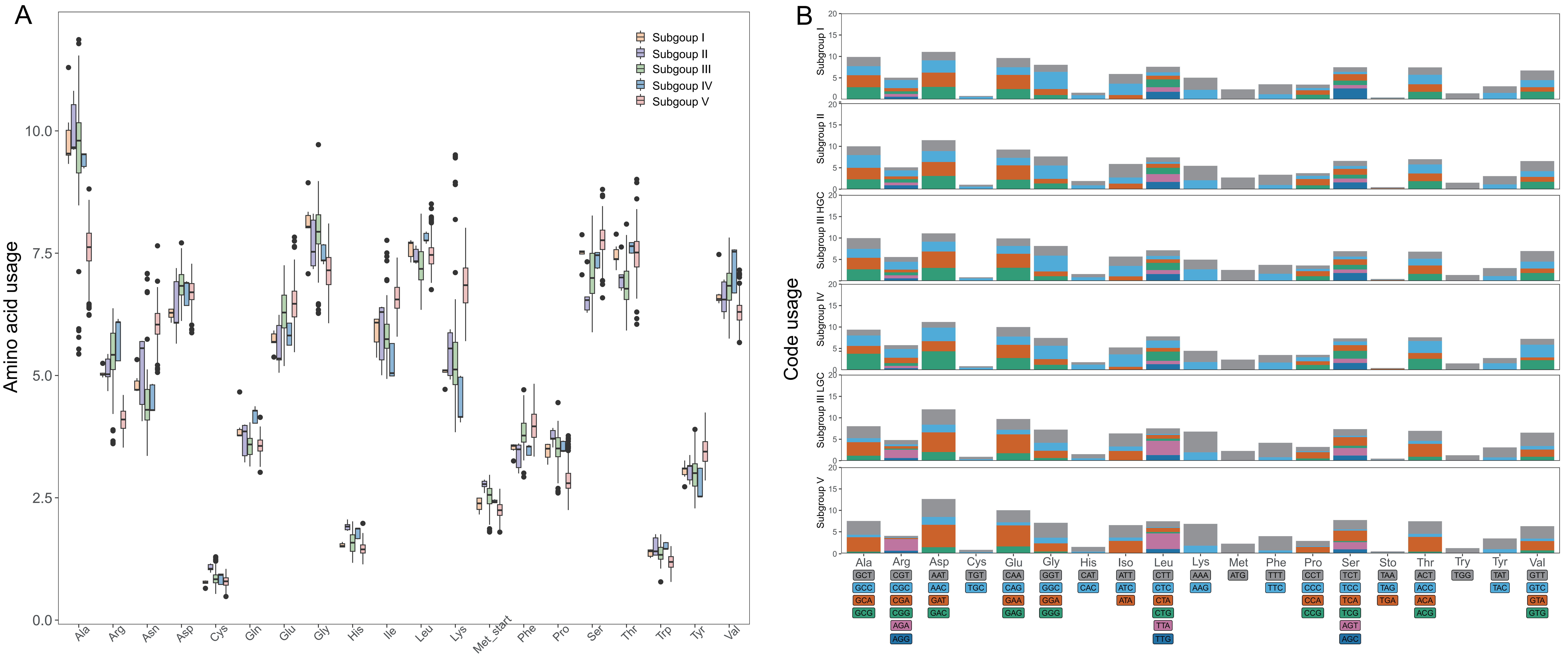

Figure S5. Comparison of amino acid and codon usage among subgroups. (A) Amino acid usage for the five subgroups. (B) Codon usage patterns for the five subgroups. Subgroup III LGC and Subgroup III HGC represent Subgroup III members with low (< 40%) and high G+C content (>40%), respectively.

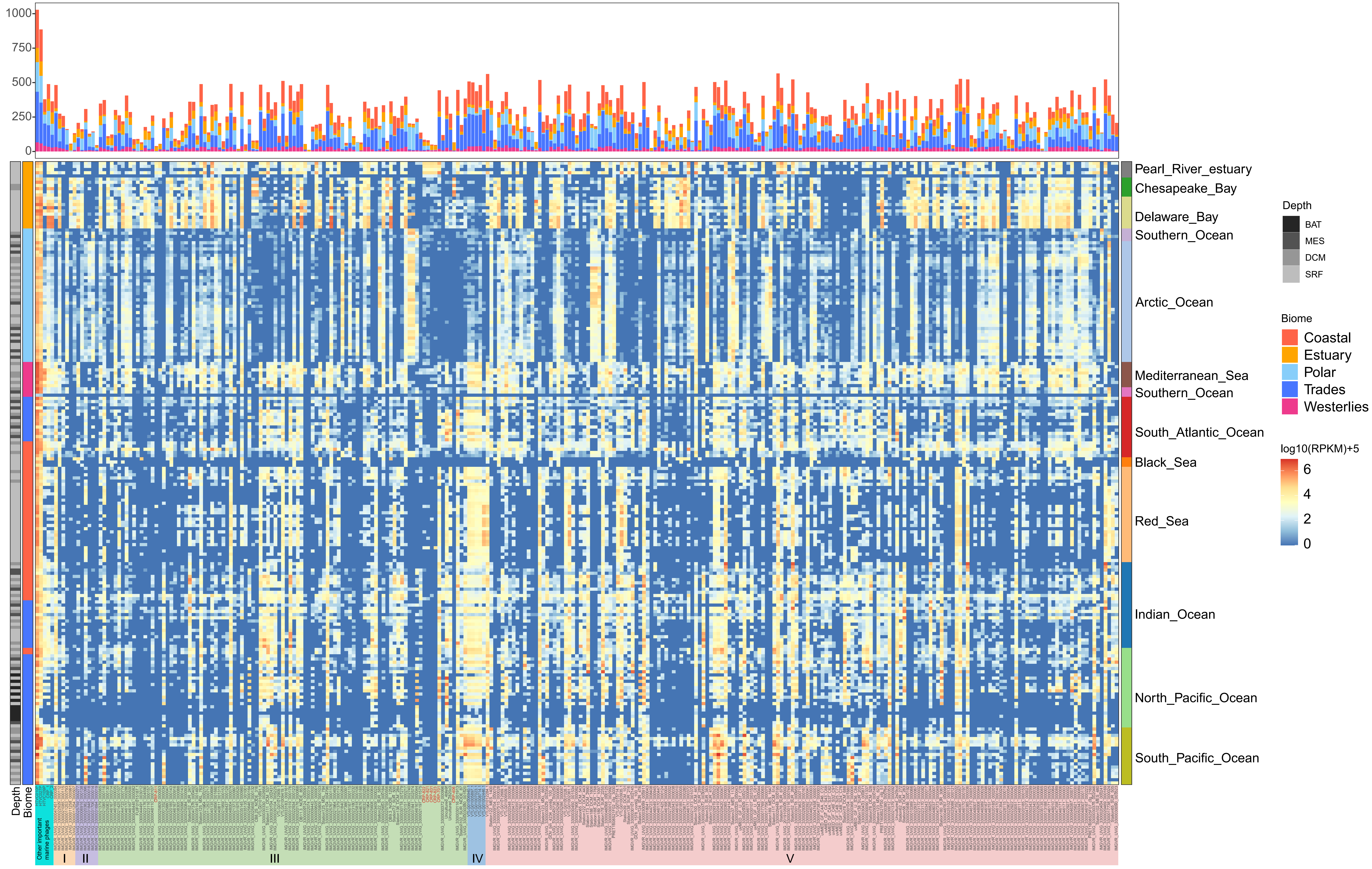

Figure S6. Heatmap of the relative abundance of CRP-822-type phages in marine viromes. The relative abundance was normalized as RPKM. The colored bars on the left indicate the biomes and depths for each station. The bar on the top shows the sum of the relative abundances of CRP-822-type phages in all samples. The colored bars on the right indicate the ocean regions or each station.
